# Prevalence, spatial distribution, and determinants of wasting among children under five in Senegal: spatial and multilevel analyses of the 2023 Senegal Demographic and Health Survey

**DOI:** 10.3389/fpubh.2025.1543945

**Published:** 2025-05-21

**Authors:** Tadesse Tarik Tamir, Berhan Tekeba, Agazhe Aemro, Mulugeta Wassie, Enyew Getaneh Mekonen

**Affiliations:** ^1^Department of Pediatrics and Child Health Nursing, School of Nursing, College of Medicine and Health Sciences, University of Gondar, Gondar, Ethiopia; ^2^Department of Medical Nursing, School of Nursing, College of Medicine and Health Sciences, University of Gondar, Gondar, Ethiopia; ^3^School of Nursing, College of Medicine and Health Sciences, University of Gondar, Gondar, Ethiopia; ^4^Department of Surgical Nursing, School of Nursing, College of Medicine and Health Sciences, University of Gondar, Gondar, Ethiopia

**Keywords:** prevalence, spatial distribution, determinants, wasting, children, Senegal

## Abstract

**Introduction:**

Child undernutrition remains a persistent public health challenge across sub-Saharan Africa. Wasting, which is characterized by severe muscle wasting and heightened vulnerability to morbidity and mortality, poses a particularly grave concern in the region. Recently, Senegal’s children faced a number of socioeconomic challenges, including the lingering effects of the COVID-19 pandemic, disruptions caused by the war between Russia and Ukraine, persistent security threats from the Sahel crisis, and internal socio-political tensions. Hence, this study aimed to assess the prevalence, spatial distribution, and determinants of wasting among children under five in Senegal.

**Method:**

A secondary analysis of the 2023 Senegal Demographic and Survey dataset was conducted. A total weighted sample of 4,392 children under the age of 5 years was included in the analysis. ArcGIS 10.7 and Stata 17 were utilized for spatial analysis and multilevel regression, respectively. The fixed effect was evaluated by calculating the adjusted odds ratio with a 95% confidence interval. We considered the association between explanatory variables and outcomes to be significant when the *p*-value was less than the predetermined level of significance (0.05).

**Result:**

The prevalence of wasting among children under the age of five in Senegal was 10.29% at a 95% CI of 9.42 to 11.24%. Childhood wasting exhibited a clustered pattern across regions of Senegal. The hot spot clusters of elevated wasting prevalence were found to be concentrated in the Diourbel, Tambacounda, and Matam regions of Senegal. Small birth size, poor household wealth index, rural residence, and region were significant determinants of wasting among children under 5 years old.

**Conclusion:**

Childhood wasting remains a significant public health concern in Senegal, with a prevalence much higher than the global 2025 target. Spatial clustering of high wasting prevalence in the Diourbel, Tambacounda, and Matam regions highlights the need for targeted interventions in these high-risk areas. The identified risk factors, including small birth size, poor household wealth, rural residence, and regional disparities, should guide the design and implementation of effective nutritional programs and policies. Addressing the underlying determinants of childhood wasting is crucial to improve child health and development outcomes in Senegal.

## Introduction

Child undernutrition remains a persistent public health challenge across sub-Saharan Africa. Wasting, the acute manifestation of malnutrition characterized by severe muscle wasting and heightened vulnerability to morbidity and mortality, poses a particularly grave concern in the region (1, 2). In 2023, an estimated 13.6 million children under five in sub-Saharan Africa were affected by wasting, accounting for over half the global burden of this life-threatening condition (3). For children under the age of five, wasting is defined as having a weight-for-height ratio that is more than 2 standard deviations below the median value in the World Health Organization (WHO) Child Growth Standards (4).

Senegal, a West African nation, has made notable progress in reducing child mortality and improving nutritional outcomes in recent decades (5–7). However, in 2023, Senegal’s children faced a number of socioeconomic challenges, including the lingering effects of the COVID-19 pandemic, disruptions caused by the war between Russia and Ukraine, persistent security threats from the Sahel crisis, and internal socio-political tensions (8). While Senegal’s economic growth rate exceeded the sub-Saharan African average, high inflation continued to impact families and children, leading to an increase in the prevalence of severe acute malnutrition from 8% in 2019 to 10.2% in 2023 (8).

Prior research has identified several factors associated with childhood wasting, including socioeconomic status, maternal education, access to healthcare, sanitation, and food security (7, 9–12). Despite these insights, gaps persist in our understanding of the regional and contextual factors underlying the ongoing public health challenge of persistent wasting across Senegal.

This study aimed to leverage the 2023 Senegal Demographic and Health Survey (SDHS) to conduct a comprehensive spatial and multilevel analysis of the prevalence, geographic patterning, and multilevel predictors of wasting among children under five in Senegal. Previous studies have primarily focused on national-level estimates, often overlooking subnational variations and the complex interplay of factors at different levels (13, 14). The 2023 SDHS provides a unique and comprehensive dataset that includes detailed geographic and multilevel data, making it particularly suited for this type of analysis. By integrating spatial epidemiological analysis with multilevel modeling techniques, this study builds on previous research by offering a more nuanced understanding of the spatial and multilevel determinants of wasting (14, 15).

Spatial analysis allows us to identify and visualize the geographic distribution of wasting at a subnational level, revealing regional hotspots and patterns that may be masked in national-level analyses. This geographic perspective is crucial for understanding the spatial heterogeneity of wasting and for designing targeted interventions that address specific regional needs. Multilevel analysis, on the other hand, enables us to examine the complex interplay of factors at different levels—individual and community—that influence a child’s nutritional status. By considering these multiple levels simultaneously, we can uncover how broader contextual factors, such as community socioeconomic status or access to healthcare, interact with individual and household characteristics to impact wasting. This comprehensive approach provides a deeper understanding of the determinants of wasting and helps identify leverage points for multisectoral strategies. The insights gained can inform the design of targeted, context-specific interventions and multisectoral strategies to address the underlying determinants of this pressing public health issue.

## Methods

### Data, sampling, and population

A secondary analysis of the 2023 Senegal Demographic and Survey dataset aimed to assess the prevalence, spatial distribution, and determinants of wasting among children under five. The DHS Program provides access to a wide range of datasets from surveys conducted across many countries. The available datasets can be accessed through their website at https://www.dhsprogram.com/data/available-datasets.cfm. The DHS data were made accessible to researchers and analysts through a formal request process managed by the MEASURE DHS program. Users can browse the available datasets and request access as needed for their research.

The Demographic and Health Survey (DHS) utilizes a robust sampling methodology to ensure the data are nationally representative. First, the country is divided into distinct strata based on relevant characteristics, such as urban/rural location or geographic regions. Within each stratum, the primary sampling units (clusters) are randomly selected, with these clusters typically comprising multiple households. In the second stage of sampling, a sample of households is drawn from within each selected cluster, using either a systematic or random approach.

Our study population consisted of all children under the age of 5 years who were residing in the sampled households. However, we excluded children who were missing key anthropometric data necessary for assessing their wasting status.

### Variables of the study

The study’s primary outcome was childhood wasting, which was assessed using the World Health Organization’s (WHO) Child Growth Standards (4). Specifically, children under 5 years of age were classified as experiencing wasting if their weight-for-height z-score was below −2 standard deviations (SD) from the WHO median (4). This measure, commonly referred to as childhood wasting, was used to evaluate the prevalence of this nutritional status indicator.

In terms of handling missing values, the researchers followed the guide to the Demographic and Health Survey (DHS) statistics. Children who were not weighed and measured, as well as those with missing weight and height values, were excluded from both the denominators and numerators. Furthermore, children flagged for out-of-range or invalid z-scores were also excluded from the denominators and numerators. This approach ensured that the analysis of childhood wasting was based on valid and reliable anthropometric data, and that the study’s findings were representative of the target population.

In this study, the researchers considered explanatory variables at two levels: individual or household variables (first level) and community (cluster) level variables (second level). The first-level variables included the child’s age, gender, birth size, maternal age, maternal educational status, maternal occupation, paternal educational status, paternal occupation, household size, wealth index, birth interval, antenatal care (ANC) visits, media exposure, type of birth, source of drinking water, and type of toilet facility. These variables were selected based on their relevance to the study’s objectives and their consistent identification in previous research as significant predictors of childhood wasting. At the second level, the researchers examined community-related factors, such as the place of residence, community illiteracy rate, community media exposure, community poverty level, and region. These variables were selected based on their potential to capture broader contextual influences on a child’s nutritional status. For instance, the place of residence (urban vs. rural) can significantly impact access to healthcare and nutritional resources. Community illiteracy rate and media exposure reflect the overall educational and informational environment, which can influence health behaviors and practices. Community poverty level indicates the economic conditions of the area, which can affect food security and access to services. The region was included to account for geographic variations in cultural, economic, and environmental factors.

### Data management

The analysis utilized the children’s recode (KR) datasets from the MEASURE DHS archive, which were extracted and loaded into Stata 17 for the formal statistical analysis. Prior to conducting the analysis, a thorough data cleaning process was undertaken. This involved checking for any duplicate records, as well as identifying and removing any implausible or invalid anthropometric measurements.

The analysis results were reported using weighted prevalence estimates and frequencies, presented in tables and bar charts. To account for the complex survey design of the DHS data, the analysis methodology incorporated the sampling weights provided in the dataset. Additionally, the standard errors of the regression coefficients were adjusted to reflect the probability of sample selection and the multistage cluster sampling approach. This ensures that the analysis estimates are representative of the target population and the statistical inferences drawn are valid.

### Spatial analysis

To investigate the spatial dependency of zero-dose childhood immunization in Ethiopia, the researchers used spatial autocorrelation techniques. Specifically, they utilized an optimized hot spot analysis method to identify statistically significant spatial clusters of high (hot spots) and low (cold spots) rates of zero-dose children.

The optimized hot spot analysis, an extension of the traditional hot spot analysis (Getis-Ord Gi* statistic), was used to detect spatial clustering patterns. This technique examines the distribution of features and their surrounding neighbors to determine if there are any statistically significant aggregations (16). Additionally, it applies the false discovery rate (FDR) correction method to adjust the results for multiple testing and spatial dependence (16, 17).

Furthermore, the researchers utilized the spatial SaTScan statistics method, which involves scanning the study area using a circular window. The geographic coordinate data, including the cases (zero-dose children) and controls (non-zero dose children), were fitted to the Bernoulli model. For each potential cluster, the researchers calculated the log likelihood ratio (LLR), relative risk (RR), and *p*-values to assess whether the observed number of cases within the cluster was significantly higher than expected. Finally, the researchers used an interpolation technique called kriging to predict the rates of zero-dose childhood immunization in unsampled areas of Ethiopia, based on the observed data from the sampled areas.

### Multilevel modeling

This study utilized data from the DHS, which contains information at both the household and cluster levels. To address the issue of non-independent observations, which is a requirement for standard logistic regression, we used mixed-effects models with a binary outcome variable. We fitted four different model specifications: a null model to assess the random effect and determine the appropriateness of multilevel regression, Model I, which included the outcome variables and first-level control variables, Model II, which incorporated the outcome variables and second-level control variables, and a final comprehensive Model III, which included all outcome variables, first-level controls, and second-level controls. By using this multilevel modeling approach, we accounted for the hierarchical structure of the DHS data and more effectively investigated the determinants of childhood wasting. The multilevel regression model was equated as follows (18):


$$\log(\frac{\mathrm{\Pi ij}}{1-\mathrm{\Pi ij}})=\beta 0+\beta 1x1\mathrm{ij}+\ldots +\text{βnxnij}+\gamma 0+\gamma 1z1\mathrm{ij}+\ldots +\text{γmzmij}+\text{uoij}$$

where πij represents the probability of wasting for the i^th^ child in the j^th^ cluster, while (1-πij) denotes the probability of the i^th^ child in the j^th^ cluster not experiencing wasting. The intercept term β0 characterizes the baseline of the regression equation. The coefficients β1 to βn are linked to the level 1 variables x1ij to xnij, which exert an influence on the response variable at the individual level. The intercept γ0j captures the random effect at level 2, while the coefficients γ1 to γm are associated with the level 2 variables z1ij to zmij, reflecting cluster-level effects. Finally, the error term eij accounts for the random error or residual within the model.

The researchers evaluated both the fixed and random components of the mixed-effects models. The random effect was assessed using the variance, intra-class correlation coefficient (ICC), median odds ratio (MOR), and proportional change in variation (PCV). The fixed effect was evaluated by calculating the adjusted odds ratio (AOR) with a 95% confidence interval (CI). We considered the association between explanatory variables and outcomes to be significant when the *p*-value was less than the predetermined level of significance (0.05). For model comparison, the researchers utilized the log likelihood, deviance, Akaike’s Information Criterion (AIC), and Bayesian Information Criterion (BIC). To address multicollinearity, which can occur when two or more independent variables in a regression model are highly correlated, the researchers calculated the variance inflation factor (VIF) for each variable and found that VIF values were below five, indicating that multicollinearity was not a significant concern. Additionally, the researchers used multivariable regression techniques to control for potential confounding factors, which are variables that might be associated with both the independent and dependent variables and could influence the observed relationship. This approach allowed the researchers to isolate the effect of each independent variable on the dependent variable while accounting for other relevant factors.

## Results

### Descriptive statistics of the study

A total weighted sample of 4,392 children under the age of 5 years was included in the analysis of this study (52.55% male children and 47.45% female children). Out of 2,308 male and 2,084 female children, 253 (11.29%) male children and 187 (9.20%) female children were wasted. Additionally, 11.06% of the 810 children born with a small size had wasting. Furthermore, 11.93% of children born to mothers with no formal education were positive for wasting. Moreover, 322 (12.36%) of the 2,684 children who were rural residents included in this study were identified as having acute malnutrition (wasting). Notably, childhood wasting was highest in the Matam region (23.13%) and lowest in the Dakar region (5.19%) of Senegal (Table 1).

**Table 1:** Descriptive statistics of the study (*n* = 4,392).

Variables		Weighted frequency (%)	Wasting status		*P*-value
			Wasted [*n* (%)]	Not wasted [*n* (%)]	
Age	Under 24 months	1,853(42.19)	181 (10.01)	1,629 (89.99)	0.93
	24 months or more	2,539 (57.81)	258 (10.50)	2,199 (89.50)	
Gender	Male	2,308 (52.55)	253 (11.29)	1985 (88.71)	0.128
	Female	2084 (47.45)	187 (9.20)	1843 (90.80)	
Birth size	Large	579 (21.17)	37 (6.66)	518 (93.34)	0.001
	Average	1,346 (49.22)	133 (10.19)	1,172 (89.81)	
	Small	810 (29.61)	88 (11.06)	706 (88.94)	
Birth interval	Less than 2 years	520 (15.81)	60 (12.01)	439 (87.99)	0.303
	Two years or more	2,767 (84.19)	265(9.81)	2,432 (90.19)	
Child is twin	Single	4,223 (96.15)	423 (10.31)	3,678 (89.69)	0.867
	Multiple	169 (3.85)	17 (9.93)	151 (90.07)	
Had diarrhea recently	No	3,388 (77.15)	341 (10.38)	2,941 (89.62)	0.867
	Yes	1,004 (22.85)	99 (10.00)	887 (90.00)	
Maternal age	15–19	190 (4.33)	23 (12.20)	165 (87.80)	0.366
	20–34	2,954 (67.26)	302 (10.53)	2,567 (89.47)	
	35–49	1,248 (28.41)	114 (9.44)	1,096 (90.56)	
Maternal education	No education	2,508 (57.10)	290 (11.93)	2,144 (88.07)	0.003
	Primary	859 (19.56)	66 (7.79)	780 (92.21)	
	Secondary or higher	1,025 (23.34)	83 (8.41)	905 (91.59)	
Maternal occupation	No working	2,900 (66.04)	312 (11.08)	2,505 (88.92)	0.064
	Working	1,491 (33.96)	127 (8.77)	1,323 (91.23)	
Paternal education	Education	3,013 (72.39)	318 (10.85)	2,611 (89.15)	0.138
	Primary	451 (10.83)	43 (9.69)	397 (90.31)	
	Secondary or higher	698 (16.78)	54 (7.99)	625 (92.01)	
Paternal occupation	Unemployed	986 (23.69)	83 (8.65)	877 (91.35)	0.490
	Employed	3,176 (76.31)	332 (10.74)	2,756 (89.26)	
ANC	No visits	324 (12.88)	31 (9.75)	287 (90.25)	0.165
	Had visits	2,194 (87.12)	210 (9.88)	1915 (90.12)	
Type of toilet facility	Improved	3,236 (73.68)	298 (9.47)	2,850 (90.53)	0.012
	Unimproved	1,156 (26.32)	141 (12.60)	978 (87.40)	
Family size	2–5	375 (8.54)	43 (11.64)	328 (88.36)	0.790
	6 or more	4,017 (91.46)	396 (10.16)	3,501 (89.84)	
Wealth index	Poorest	1,018 (23.18)	133 (13.47)	856 (86.53)	0.001
	Poorer	972 (22.12)	107 (11.36)	835 (88.64)	
	Middle	913 (20.79)	99 (11.03)	798 (88.97)	
	Richer	792 (18.04)	54 (7.02)	710 (92.98)	
	Richest	697 (15.87)	46 (6.87)	629 (93.13)	
Media exposure	No	1,544 (35.16)	173 (11.50)	1,330 (88.50)	0.352
	Yes	2,847 (64.84)	266 (9.64)	2,498 (90.36)	
Source of drinking water	Improved	3,646 (83.02)	334 (9.42)	3,212 (90.58)	0.040
	Unimproved	746 (16.98)	105 (14.59)	616 (85.41)	
Residence	Urban	1,708 (38.89)	118 (7.07)	1,549 (92.93)	0.004
	Rural	2,684 (61.11)	322 (12.36)	2,280 (87.64)	
Community illiteracy rate	Low	2,192 (49.91)	159 (7.46)	1976 (92.54)	0.001
	High	2,200 (50.09)	280 (13.13)	1852 (86.87)	
Community media utilization	Low	2,022 (46.05)	205 (10.48)	1752 (89.52)	0.129
	High	2,369 (53.95)	234 (10.13)	2077 (89.87)	
Community poverty level	Low	2,505 (57.03)	196 (8.03)	2,241 (91.97)	0.001
	High	1,887 (42.97)	244 (13.30)	1,587 (86.70)	
Region	Dakar	835 (19.01)	43 (5.19)	784 (94.81)	0.001
	Ziguinchor	133 (3.02)	8 (5.88)	125 (94.12)	
	Diourbel	522 (11.89)	88 (17.82)	404 (82.18)	
	Saint-Louis	361 (8.22)	29 (8.32)	322 (91.68)	
	Tambacounda	215 (4.89)	39 (18.65)	168 (81.35)	
	Kaolack	340 (7.75)	32 (9.72)	297 (90.28)	
	Thies	588 (13.39)	36 (6.36)	533 (93.64)	
	Louga	251 (5.72)	27 (11.09)	213 (88.91)	
	Fatick	278 (6.34)	32 (11.83)	238 (88.17)	
	Kolda	221 (5.03)	22 (9.90)	196 (90.10)	
	Matam	195 (4.43)	43 (23.13)	141 (76.87)	
	Kaffrine	257 (5.86)	22 (8.67)	234 (91.33)	
	Kedougou	58 (1.31)	5 (7.94)	53 (92.06)	
	Sedhiou	138 (3.13)	16 (11.53)	119 (88.47)	

ANC, antenatal care.

The chi-square association testing of this study revealed that a total of nine variables were distinctly associated with wasting (Table 1). These variables were size at birth, maternal education, type of toilet facility, source of drinking water, residence, community illiteracy rate, community poverty level, and region.

### Prevalence of wasting among children under five in Senegal

**Figure 1:**
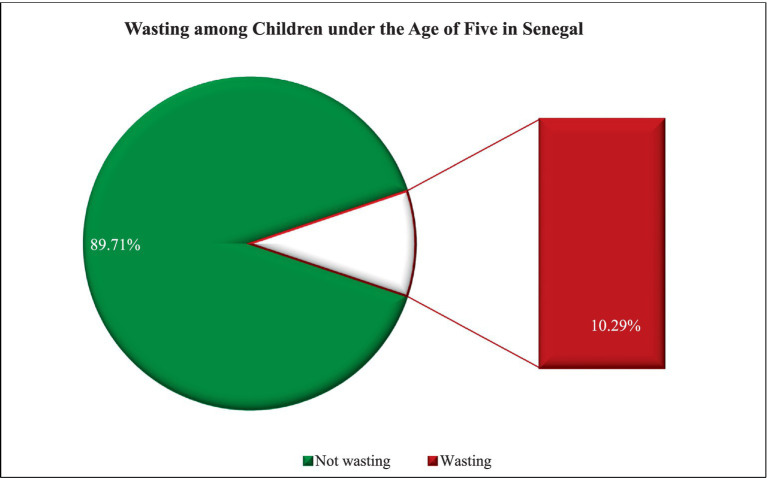
Prevalence of wasting among children under the age of five in Senegal, DHS 2023.

The national prevalence of wasting among children under the age of five in Senegal was 10.29% at a 95% confidence interval of 9.42 to 11.24% (Figure 1).

### Spatial autocorrelation

**Figure 2:**
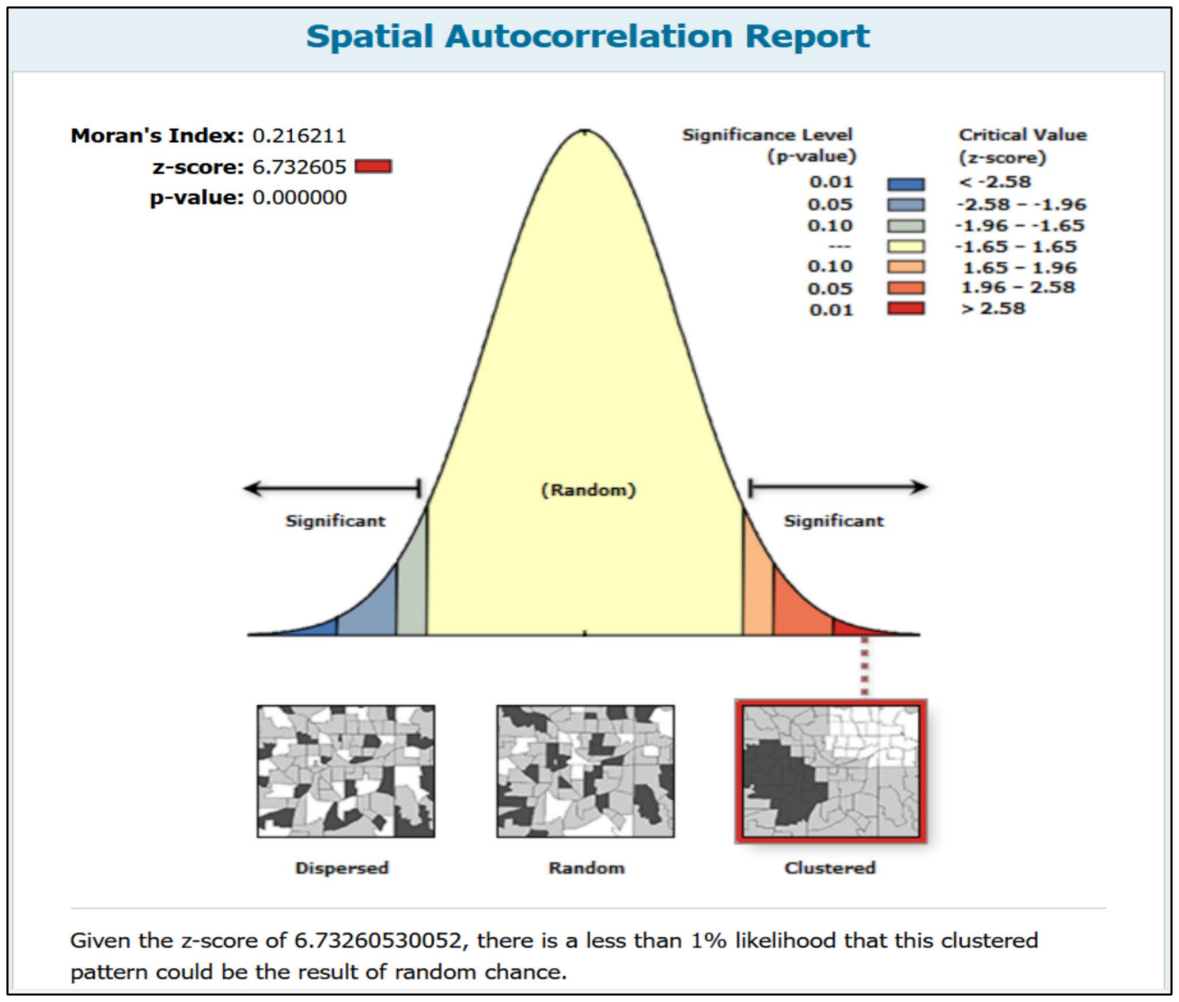
Spatial autocorrelation of wasting among children under the age of five in Senegal, DHS 2023.

The spatial autocorrelation analysis showed a positive Moran’s Index of 0.216211, indicating a degree of positive spatial autocorrelation. In other words, the high positive Z-score of 6.732605 suggests that the observed spatial clustering of wasting among children is significantly different from what would be expected under a random distribution. Furthermore, the very low *p*-value of less than 0.0001 confirms the statistical significance of this spatial clustering. These results demonstrate that childhood wasting is not randomly distributed across the region, but rather exhibits a clustered pattern. This means that areas with similar wasting prevalence tend to be located in proximity to one another (Figure 2).

### Hot spot analysis of wasting among children under the age of five in Senegal

**Figure 3:**
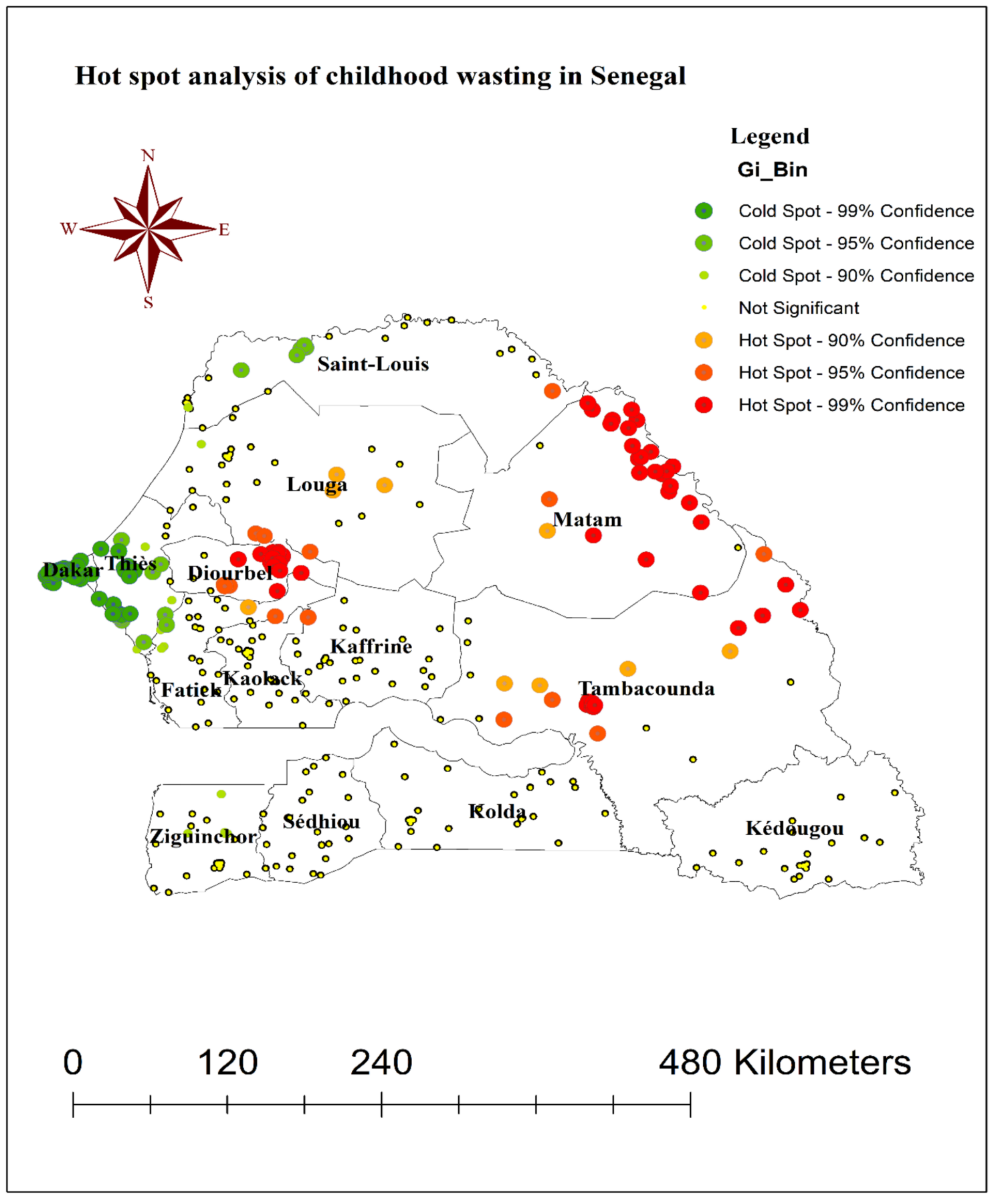
Hot spot analysis of wasting among children under the age of five in Senegal, DHS 2023.

The hot spot analysis conducted as part of this study identified the locations of high-risk “hot spot” clusters and low-risk “cold spot” clusters for childhood wasting across Senegal. As shown in Figure 3, the red points on the map indicate the areas where hot spot clusters of wasting were detected, while the green points denote the locations of cold spot clusters.

Specifically, the hot spot clusters of elevated wasting prevalence were found to be concentrated in the Diourbel, Tambacounda, and Matam regions of Senegal. This suggests that these geographic areas are experiencing particularly high levels of childhood wasting compared to other parts of the country. In contrast, the cold spot clusters, representing regions with significantly lower wasting rates, were observed in the Dakar, Thies, and western Saint-Louis areas. These regions can be considered relatively “low-risk” zones for childhood wasting within Senegal.

### SaTScan analysis of wasting among children under the age of five in Senegal

The SaTScan spatial cluster analysis, which was conducted using 30% of the total population, identified two statistically significant cluster windows for childhood wasting in Senegal—one primary cluster and one secondary cluster.

The most likely (primary) cluster window was located at 14.749142°N latitude and 15.701673°W longitude, with a radius of 18.72 km. The children living within this primary cluster window were found to have a 3.88 times higher risk of wasting compared to children outside the cluster. Additionally, a secondary cluster window was detected at 15.030375°N latitude and 13.659316°W longitude, with a larger radius of 191.77 km. The children residing within this secondary cluster window had a 2.20 times higher risk of wasting relative to those outside the cluster (Table 2).

**Table 2:** SaTScan analysis of wasting among children under the age of five in Senegal, DHS 2023.

Cluster	N	Latitude	Longitude	Radius (km)	Population	Cases	RR	LLR	*p*-value
Primary	4	14.749142 N	15.701673 W	18.72	104	39	3.88	27.85	<0.001
Secondary	69	15.030375 N	13.659316 W	191.77	595	116	2.20	26.51	<0.001

**Figure 4:**
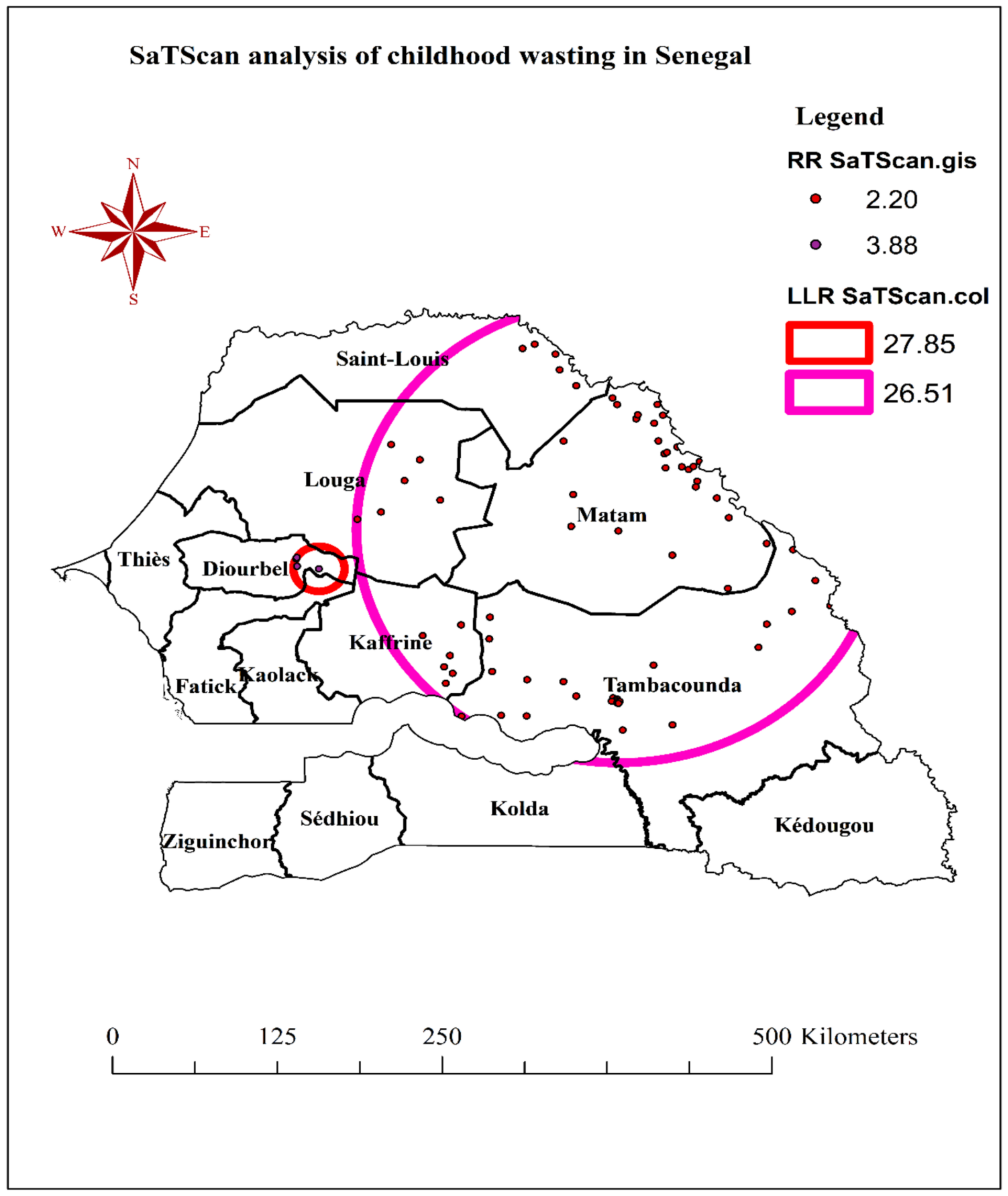
SaTScan analysis of wasting among children under the age of five in Senegal, DHS 2023.

While the primary cluster window encircled areas in the eastern parts of the Diourbel region and the northeast border of the Fatick region, the secondary cluster window encompassed the entire Matam, Tambacounda, and southeastern portions of the Kaffrine, Louga, and Saint-Louis regions (Figure 4).

### Spatial interpolation

**Figure 5:**
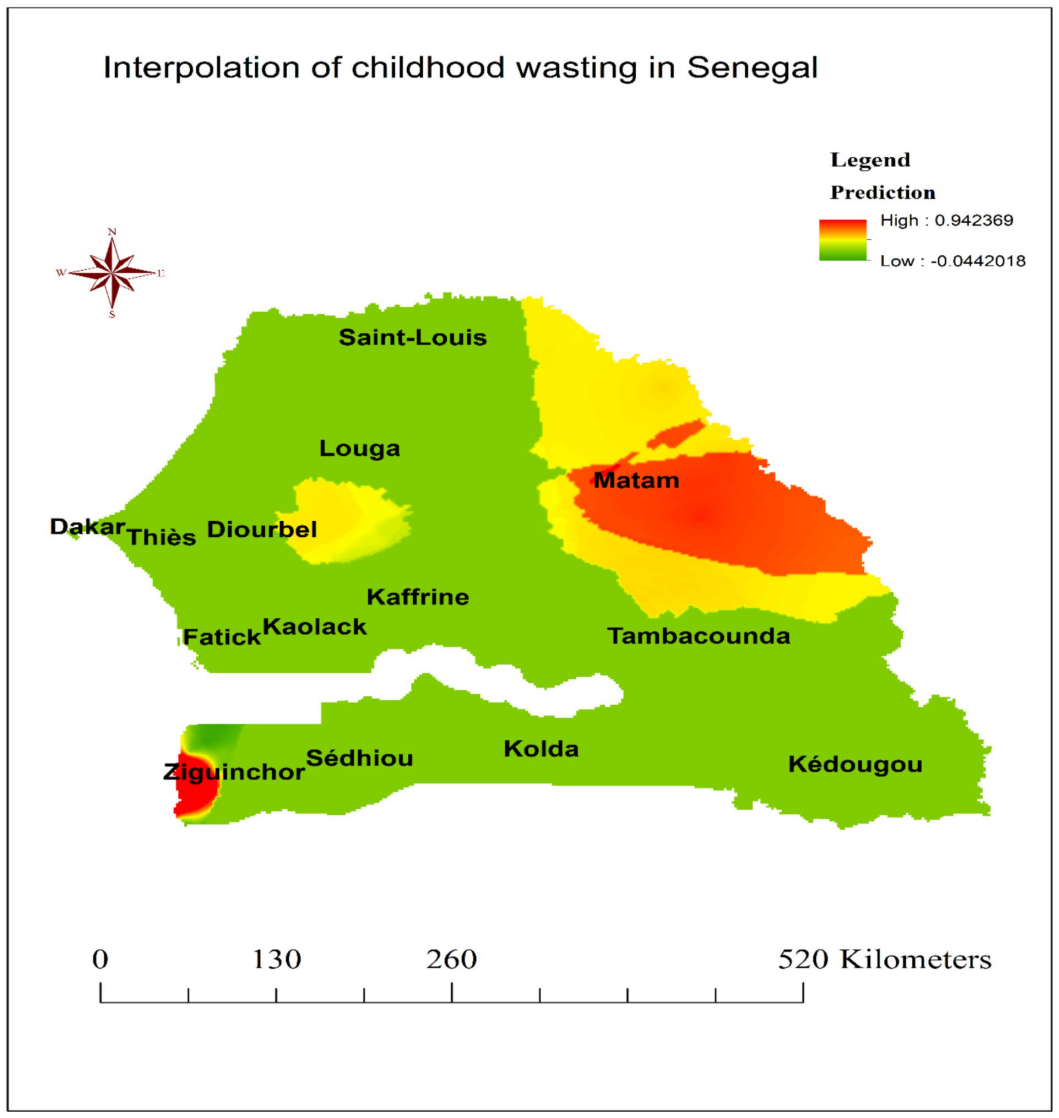
Kriging interpolation of wasting among children under the age of five in Senegal, DHS 2023.

The ordinary kriging interpolation map shown in Figure 5 displays the predicted spatial distribution of childhood wasting across Senegal. The red shaded areas on the map indicate regions with high predicted values of wasting prevalence, while the green areas correspond to locations with lower predicted wasting proportions. Based on the kriging analysis, it was found that the majority of the Matam region and the western portion of the Ziguinchor region were predicted to have high proportions of wasting among children under 5 years of age (Figure 5).

### Random effect (measures of variation) and model fitness

The results from your multilevel models examining the prevalence, spatial distribution, and determinants of wasting among children under 5 years old in Senegal provide valuable insights. Decreasing random effect variance from the null Model 0 to the full Model III indicates that the inclusion of explanatory variables in the subsequent models helps explain a substantial portion of the between-cluster (community) variability in the risk of child wasting. This reduction in random effect variance from Model 0 to Model III is 67.53%, suggesting that the variables included in the final Model III account for a large proportion of the initial unexplained between-cluster heterogeneity in child wasting. Additionally, the intra-class correlation (ICC) decreases from 10.72% in the null Model 0 to 3.75% in the full Model III, implying that a smaller proportion of the total variance in child wasting is attributable to between-cluster differences after accounting for the explanatory variables in the model. Such a finding suggests that the individual- and cluster-level factors included in the models help explain a significant portion of the contextual influences on child wasting.

Furthermore, the decreasing median odds ratio (MOR) from 1.82 in the null Model 0 to 1.41 in the full Model III indicates that the unexplained between-cluster heterogeneity in the log odds of child wasting is reduced by the inclusion of the explanatory variables. This means that, on average, the child’s odds of wasting would increase by a factor of 1.41 if they were to move from a cluster (community) with a lower risk to a cluster with a higher risk, after accounting for the individual- and cluster-level factors in the final Model III. Finally, the improvements in model fit statistics, such as the increasing log-likelihood (LL) values, decreasing deviance, and lower AIC and BIC values from the null Model 0 to the full Model III, further confirm that the inclusion of the explanatory variables significantly enhances the model’s ability to explain the determinants of wasting among children under 5 years old in Senegal (Table 3).

**Table 3:** Random effect and model fitness of wasting among children under the age of five in Senegal, DHS 2023.

Parameters	Model 0	Model I	Model II	Model III
Random effect				
Variance	0.3951249	0.3258096	0.1490077	0.1282953
ICC	0.1072254	0.0901102	0.0433303	0.0375
MOR	1.82	1.72	1.44	1.41
PCV	Reference	17.54%	62.29	67.53%
Model comparison				
LL	−1548.27	−903.30	−1506.19	−879.09
Deviance	3,096.54	1,806.60	3,012.38	1,758.18
AIC	3100.54	1830.602	3048.38	1814.192
BIC	3113.35	1980.07	3163.74	1901.69

AIC, Akaike’s Information Criterion; BIC, Bayesian Information Criterion; ICC, intra-class correlation coefficient; MOR, median odds ratio, LL, log likelihood; PCV, proportional change in variance.

### Measures of association (fixed effect)

In our multivariable multilevel regression analysis, we aimed to construct a parsimonious model that captured the key determinants of the outcome variable, wasting. To achieve this, we first conducted a preliminary bivariable regression analysis to identify factors that had a *p*-value less than 0.25. This less stringent *p*-value threshold was chosen to ensure that potentially important variables were not prematurely excluded from the multivariable modeling stage. Notably, we found that only factors that had demonstrated statistically significant associations with wasting in the initial chi-square tests had *p*-values below 0.25 in the bivariable regressions. This suggested that these variables were indeed strongly associated with the outcome of interest and merited inclusion in the multivariable model.

Accordingly, the findings from the fixed effects portion of the multilevel regression analysis revealed that birth size, household wealth index, place of residence, and region were significant determinants of wasting among children under 5 years old in Senegal.

The odds ratio of 1.55 (95% CI: 1.16, 2.08) for the “small birth size” variable indicates that children with a small birth size had 55% higher odds of being wasted compared to children with an average birth size, after accounting for other factors in the model. Similarly, the odds ratio of 2.28 (95% CI: 1.20, 4.36) for the “poorest household wealth index” category shows that children from the poorest households have more than two times the odds of being wasted compared to their counterparts from wealthier households.

Furthermore, the odds ratio of 1.20 (95% CI: 1.05, 1.92) for “rural residence” suggests that children living in rural areas have 20% higher odds of being wasted relative to their urban counterparts.

The regional differences in child wasting are quite pronounced. Compared to the reference category of the Dakar region, children living in the Diourbel, Tambacounda, and Matam regions have substantially higher odds of wasting, with odds ratios of 4.06 (95% CI: 1.60, 10.29), 3.35 (95% CI: 1.31, 8.52), and 4.92 (95% CI: 1.95, 12.40), respectively (Table 4).

**Table 4:** Measures of association of wasting among children under the age of 5 years in Senegal, DHS 2023.

Factors		Model I AOR at 95% CI	Model II AOR at 95% CI	Model III AOR at 95% CI
Birth size	Large	0.67 (0.46, 0.97)		0.70 (0.48, 1.01)
	Average	1.00		1.00
	Small	1.47 (1.10, 1.96)		1.55 (1.16, 2.08) *
Maternal education	No education	1.22 (0.86, 1.72)		1.05 (0.73, 1.51)
	Primary	1.10 (0.73, 1.67)		1.11 (0.74, 1.68)
	Secondary or higher	1.00		1.00
Type of toilet facility	Improved	1.00		1.00
	Unimproved	1.31 (0.95, 1.83)		1.22 (0.87, 1.69)
Wealth index	Poorest	2.38 (1.26, 4.52)		2.28 (1.20, 4.36) *
	Poorer	2.36 (1.24, 4.51)		1.92 (0.97, 3.79)
	Middle	1.94 (1.01, 3.72)		1.76 (0.91, 3.40)
	Richer	1.62 (0.81, 3.22)		1.62 (0.81, 3.22)
	Richest	1.00		1.00
Source of drinking water	Improved	1.00		1.00
	Unimproved	1.25 (0.91, 1.72)		1.18 (0.84, 1.65)
Residence	Urban		1.00	1.00
	Rural		1.22 (1.08, 1.95)	1.20 (1.05, 1.92) *
Community illiteracy rate	Low		1.00	1.00
	High		1.27 (0.98, 1.64)	1.26 (0.79, 1.99)
Community poverty level	Low		1.00	1.00
	High		1.30 (0.96, 1.75)	1.26 (0.79, 1.99)
Region	Dakar		1.00	1.00
	Ziguinchor		1.25 (0.53, 2.97)	1.44 (0.46, 4.53)
	Diourbel		3.41 (1.70, 6.84)	4.06 (1.60, 10.29) *
	Saint-Louis		1.59 (0.76, 3.32)	1.42 (0.52, 3.88)
	Tambacounda		3.41 (1.71, 6.82)	3.35 (1.31, 8.52) *
	Kaolack		1.78 (0.85, 3.72)	1.81 (0.66, 5.00)
	Thies		1.33 (0.63, 2.82)	1.55 (0.58, 4.10)
	Louga		2.22 (1.08, 4.55)	2.13 (0.81, 5.64)
	Fatick		2.24 (1.10, 4.55)	2.45 (0.94, 6.42)
	Kolda		1.73 (0.82, 3.64)	1.69 (0.62, 4.62)
	Matam		5.29 (2.68, 10.46)	4.92 (1.95, 12.40) *
	Kaffrine		1.31 (0.63, 2.73)	1.39 (0.52, 3.71)
	Kedougou		1.60 (0.75, 3.44)	1.22 (0.42, 3.49)
	Sedhiou		1.94 (0.93, 4.04)	2.24 (0.83, 6.04)

AOR, adjusted odds ratio; CI, confidence interval; *Statistically significant at *p*-value <0.05.

## Discussion

Child undernutrition remains a persistent public health challenge across sub-Saharan Africa. Wasting, the acute manifestation of malnutrition characterized by severe muscle wasting and heightened vulnerability to morbidity and mortality, poses a particularly grave concern in the region. Recently, Senegal’s children faced a number of socioeconomic challenges, including the lingering effects of the COVID-19 pandemic, disruptions caused by the war between Russia and Ukraine, persistent security threats from the Sahel crisis, and internal socio-political tensions. Hence, this study revealed the prevalence, spatial distribution, and determinants of wasting among children under five in Senegal.

The prevalence of wasting, a form of acute undernutrition, among children under five in Senegal is alarmingly high at 10.29%, with a 95% confidence interval ranging from 9.42 to 11.24%. This finding is consistent with the reported prevalence of severe acute malnutrition among children in Senegal in 2023, which was 10.2%, according to UNICEF (8). This indicates that approximately 10 out of every 100 children in Senegal are affected by wasting, a critical situation when compared to neighboring countries. For instance, the Gambia reported a wasting prevalence of 5.9% in its 2019–2020 Demographic and Health Survey (DHS) (19), while a systematic review in 2019 found a pooled wasting prevalence of 6.1% in Guinea-Bissau (20). Looking at a broader African context, Nigeria’s 2018 DHS showed a wasting prevalence of 6.5% among children under five (21).

Furthermore, Senegal’s wasting prevalence is significantly higher than the global average of 6.8% (22) and the sub-Saharan African regional average of 7.09% (23), underscoring the country’s struggle with childhood undernutrition. This challenge is a reflection of Senegal’s challenging economic landscape, where food scarcity is a grim reality for many families (1). Cultural dietary practices, which may not always align with the nutritional needs of children, could also contribute to the issue of undernutrition in the country (24). Additionally, poor sanitation and hygiene can exacerbate health problems, leading to a vicious cycle of disease and malnutrition (25). The finding also emphasizes the need for continuous monitoring and evaluation of both the prevalence of wasting and the effectiveness of intervention programs in Senegal. International aid, when effectively managed, can play a crucial role in supplementing the country’s efforts to improve the nutritional status of its children (26).

The observation that childhood wasting is not randomly distributed but exhibits a clustered pattern is an important finding. This suggests that there are underlying geographic, socioeconomic, or environmental factors that contribute to the spatial heterogeneity of wasting prevalence. Previous studies have also shown that the distribution of malnutrition, including wasting, is often spatially correlated (27, 28). Additionally, the identification of hot spot clusters of elevated wasting prevalence in the Diourbel, Tambacounda, and Matam regions of Senegal is particularly noteworthy. This could indicate the presence of region-specific risk factors that contribute to higher rates of wasting in these areas. Some potential factors that have been linked to spatial clustering of wasting in other contexts include access to healthcare services, sanitation and hygiene, food security, and household wealth (29, 30). Moreover, the primary cluster window encircling areas in the eastern Diourbel region and the northeast border of the Fatick region, as well as the secondary cluster window encompassing the Matam, Tambacounda, and southeastern portions of the Kaffrine, Louga, and Saint-Louis regions, provided valuable spatial information to guide targeted interventions. By identifying these high-risk areas, policymakers and public health officials can prioritize resource allocation and tailor prevention and treatment strategies to address the specific needs of these communities (30). Furthermore, the Kriging interpolation, which predicted areas with high proportions of wasting, reinforces the need for a geographically informed approach to addressing childhood wasting in Senegal. This suggests that large swaths of these areas may be experiencing high wasting rates. Kriging interpolation, a geostatistical method, leverages spatial correlations in observed data to estimate the prevalence of conditions like wasting in unsampled areas (31). In this study, the technique revealed elevated wasting rates across much of the Matam region and western Ziguinchor, underscoring the need for prioritized resource allocation in these areas.

While our study provides valuable insights into the determinants of wasting, it is important to acknowledge their nature inherent in its cross-sectional design. This design restricts our ability to infer causality between the observed variables and children’s wasting. Consequently, the following associations identified through our analysis should be interpreted with caution, as they may not necessarily reflect causal relationships.

Birth size was identified as a significant predictor of wasting. Children with a small birth size had higher odds of being wasted compared to those with an average birth size, even after accounting for other factors in the model. This aligns with existing evidence that low birth weight and small birth size are strongly associated with an increased risk of acute malnutrition in early childhood (32, 33). Small birth size may be a proxy for intrauterine growth restriction, which can compromise a child’s nutrient stores and metabolic programming, predisposing them to wasting (34). The finding that children with a small birth size had higher odds of being wasted underscores the critical importance of maternal and fetal health during the prenatal period. Small birth size can be a marker of intrauterine growth restriction, which can have long-lasting consequences on a child’s physical and cognitive development (35). This points to the need for interventions that target the prevention and management of low birth weight and intrauterine growth retardation, such as improving access to quality antenatal care, addressing maternal undernutrition, and promoting optimal maternal nutrition and health behaviors (36).

Household wealth was also a significant determinant, with children from the poorest households having more than twice the odds of being wasted compared to their counterparts from wealthier households. This finding is consistent with a large body of research demonstrating the critical role of household socioeconomic status in child nutritional outcomes (14, 37–39). Poverty is closely linked to food insecurity, inadequate access to healthcare, and suboptimal child care practices, all of which increase the risk of acute malnutrition. Addressing the root causes of household poverty, such as limited access to livelihood opportunities, social protection, and essential services, may be crucial in reducing the burden of acute malnutrition among children from disadvantaged backgrounds.

Furthermore, the analysis revealed a significant rural–urban disparity, with children living in rural areas having 20% higher odds of wasting compared to their urban peers. This aligns with prior studies in sub-Saharan Africa that have identified place of residence as a key determinant of child nutritional status (40, 41). The observed rural–urban disparity in child wasting highlights the persistent geographic inequities in nutrition and health outcomes. This gap likely reflects the complex interplay of factors, including differences in access to nutritious foods, healthcare services, water and sanitation infrastructure, and caregiving practices between rural and urban areas (41). Targeted, context-specific interventions that address the unique challenges faced by rural communities, such as investing in rural infrastructure, strengthening community-based health and nutrition services, and promoting household food security, may help to narrow this divide.

The analysis uncovered pronounced regional variations in child wasting within Senegal. Compared to the Dakar region, children living in Diourbel, Tambacounda, and Matam regions had significantly higher odds of being wasted. These regional disparities underscore the importance of considering subnational differences in the design and implementation of nutrition programs. The disparities may be driven by several socioeconomic factors. Regions such as Diourbel, Tambacounda, and Matam often experience higher levels of poverty, lower household wealth, and limited access to economic opportunities compared to urban areas such as Dakar (42). These economic challenges can lead to food insecurity and inadequate access to nutritious food, contributing to higher rates of wasting (43). Additionally, rural areas may have less access to healthcare services, which can affect the timely diagnosis and treatment of malnutrition (44). Cultural practices and beliefs also play a significant role in regional disparities. In some regions, traditional dietary practices and food taboos may limit the variety and nutritional quality of food available to children (45). For example, certain foods that are rich in essential nutrients might be avoided due to cultural beliefs, impacting the nutritional status of children (43). Furthermore, practices related to infant and young child feeding, such as the duration of breastfeeding and the introduction of complementary foods, can vary significantly across regions and influence wasting rates (45). Variations in climate and agricultural productivity can also contribute to regional disparities. Regions with less favorable climatic conditions may experience lower agricultural yields, leading to food shortages and higher food prices1. This can exacerbate food insecurity and increase the risk of wasting among children. The capacity of local health systems to address malnutrition can vary widely between regions. Areas with limited healthcare infrastructure and resources may struggle to provide adequate nutrition services, such as growth monitoring, nutritional counseling, and treatment for malnutrition (42, 44). Strengthening health systems in these regions is crucial for improving child nutritional outcomes.

Taken together, the findings of this study emphasize the multifaceted and context-specific nature of child undernutrition in Senegal. Addressing this persistent public health challenge will require a comprehensive, multisectoral approach that tackles the individual, household, and community-level drivers of wasting. This may involve integrating nutrition-specific interventions, such as the promotion of optimal infant and young child feeding practices, with nutrition-sensitive approaches that address the broader social, economic, and environmental determinants of child health and wellbeing (1, 46).

The key limitations of this study are related to its cross-sectional design. As a survey conducted at a single point in time, the study is unable to establish causal relationships between the determinants and the prevalence of wasting among children under five in Senegal. The geographical coverage of the survey may also be a limiting factor, as the spatial analysis may not be fully representative of the entire country. Furthermore, the study may not have accounted for all relevant confounding factors that could influence wasting, and the 2023 data may not reflect the current situation, limiting the temporal relevance of the findings. Finally, the generalizability of the results to other contexts outside of Senegal may be limited due to the country-specific nature of the study.

## Conclusion

Childhood wasting remains a significant public health concern in Senegal, with a prevalence much higher than the global 2025 target. Spatial clustering of high wasting prevalence in the Diourbel, Tambacounda, and Matam regions highlights the need for targeted interventions in these high-risk areas. The identified risk factors, including small birth size, poor household wealth, rural residence, and regional disparities, should guide the design and implementation of effective nutritional programs and policies. Addressing the underlying determinants of childhood wasting is crucial to improve child health and development outcomes in Senegal. Continued monitoring, multisectoral collaboration, and evidence-based strategies are necessary to effectively reduce the burden of wasting and promote optimal nutrition among children under five, with a priority on addressing the geographic and socioeconomic inequities in wasting prevalence to ensure equitable progress nationwide.

### Recommendation

#### Targeted nutritional interventions

These interventions implement community-based nutritional programs focusing on high-risk regions, such as Diourbel, Tambacounda, and Matam. These programs should include supplementary feeding, growth monitoring, and nutrition education for caregivers.

#### Economic support initiatives

These initiatives develop and promote income-generating activities and social protection programs to improve household wealth, particularly in rural areas. This can help alleviate poverty and improve access to nutritious food.

#### Maternal and child health services

These services strengthen antenatal care (ANC) services to ensure early identification and management of at-risk pregnancies and promote maternal education on infant and young child feeding practices.

#### Water, sanitation, and hygiene (WASH) programs

These programs improve access to clean drinking water and sanitation facilities to reduce the risk of infections that contribute to wasting and implement community-led total sanitation (CLTS) programs to promote hygiene practices.

#### Multisectoral collaboration

It fosters collaboration between health, agriculture, education, and social protection sectors to address the multifaceted determinants of wasting and establishes a national task force to coordinate efforts and monitor progress.

#### Monitoring and evaluation

Monitoring and evaluation establish robust monitoring and evaluation systems to track the effectiveness of interventions and adjust strategies as needed and use data from the 2023 SDHS to inform policy decisions and program designs.

## Data Availability

Publicly available datasets were analyzed in this study. This data can be found at: https://www.dhsprogram.com/data/available-datasets.cfm.
